# Notes of Cases Treated in the Ophthalmic Hospital, Canton, China

**Published:** 1856-12

**Authors:** Jno. G. Kerr


### *Notes of Cases treated in the Ophthalmic Hospital, Canton, China.*

By Jno. G. Kerr, M. D.

*Case* 1*st.*—A man was admitted January 23d, having his perineum severely contused from a fall; retention of urine thirty to thirty-six hours. Several attempts to introduce the catheter failed, but caused, each time, blood to flow from the penis in a stream. The warm bath and other measures gave no relief. An incision was made in the perineum at the seat of injury to prevent sloughing, and an effort was made to puncture the bladder above the pubis, but this was stoutly resisted by the patient. On the next day, however, his sufferings compelled him to yield, and about five pints of urine were drawn off through the canula.

The instrument was retained in its place and the bladder relieved by it daily until February 2d, when for the first time a little urine was discharged from the wound in the perineum, which, with the contusion, had in the mean time almost entirely healed.

Feburary 4th. Urine discharged by natural passage and canula removed.

February 9th. Most of the urine comes away by urethra, but a little is passed from the wound in the perineum, which remained a small fistula when the patient left the Hospital.

After recovering from the shock of the fall and subsequent sufferings from retention, there was no unfavorable symptom to excite attention. The wound made by the trochar healed readily after the canula was removed. What seems singular in this case is the fact that the neck of the bladder should remain closed so long as to allow the lacerated urethra and contused perineum to return so nearly to a healthy state before the irritating urine was admitted to them.

*Case 2d.*—A man æt. 58, from the Province of Kevong Lái, has had phymosis forty-five years. During this time a concretion has formed within the prepuce. On performing the usual operation, a stone of the size and shape of the glans penis dropped out. It rested upon the glans, which, with the mucous lining of the prepuce, was healthy. The concretion has about the specific gravity and appearance of chalk, but is not perfectly white. Weight two drachms.

*Case 3d.*—A patient presented himself, May 28th, 1856, with phymosis and hypertrophy of the prepuce. Pressure gave a very peculiar sensation to the fingers. Upon opening the prepuce one hundred and three small calculi were taken out; they were smooth, of a slightly yellow color; some about the size of a pea, some larger and others smaller; weight of the whole a little more than two drachms.

*Case 4th.*—This case was similar to the last. The prepuce contained twenty-five calculi, but some of them were much larger; weight of the whole the same as last.

*Case 5th.*—A boy æt. 7, came in September 1st, with malformation of the penis. The organ had the appearance of having been split open on its dorsal surface, up to the symphisis pubis, and the deformity was found to consist, in fact, in the want of union of the corpora cavernosa of the two sides. The mucous membrane of the urethra formed a groove on the upper surface of the penis, and was continuous on both sides with the skin which covered the corpora cavernosa. The two halves of the glans seemed fully developed, and the part of the urethra between them had assumed the appearance of skin. A fold of skin at the symphisis pubis formed the upper boundary of the urethra, where it became a perfect canal. The organ became a erect while handled; when non-erect, it was drawn back, and the only part visible was the glans. When drawn out the opening of the urethra could be stretched so as to resemble somewhat the vagina of a female child. The scrotum and testicles were normally developed.

The Chinese do not seem to be subject to deformities more than people of other nations, but I have seen a considerable number of cases of hare-lip and cleft-palate, and persons with supernumerary fingers and toes are not uncommon.

